# Regulation of Nrf2/ARE Pathway by Dietary Flavonoids: A Friend or Foe for Cancer Management?

**DOI:** 10.3390/antiox9100973

**Published:** 2020-10-11

**Authors:** Tharindu L. Suraweera, H. P. Vasantha Rupasinghe, Graham Dellaire, Zhaolin Xu

**Affiliations:** 1 Department of Plant, Food, and Environmental Sciences, Faculty of Agriculture, Dalhousie University, Truro, NS B2N 5E3, Canada; th992644@dal.ca; 2 Department of Pathology, Faculty of Medicine, Dalhousie University, Halifax, NS B3H 4R2, Canada; dellaire@dal.ca (G.D.); zhaolin.xu@nshealth.ca (Z.X.); 3 Division of Anatomical Pathology and Cytopathology, QEII Health Sciences Centre, Nova Scotia Health Authority, Halifax, NS B3H 1V8, Canada

**Keywords:** cancer chemoprevention, cancer promotion, polyphenols, oxidative homeostasis, Keap1/Nrf2

## Abstract

The nuclear factor erythroid 2-related factor 2 (Nrf2)/antioxidant response element (ARE) pathway is an important cell signaling mechanism in maintaining redox homeostasis in humans. The role of dietary flavonoids in activating Nrf2/ARE in relation to cancer chemoprevention or cancer promotion is not well established. Here we summarize the dual effects of flavonoids in cancer chemoprevention and cancer promotion with respect to the regulation of the Nrf2/ARE pathway, while underlying the possible cellular mechanisms. Luteolin, apigenin, quercetin, myricetin, rutin, naringenin, epicatechin, and genistein activate the Nrf2/ARE pathway in both normal and cancer cells. The hormetic effect of flavonoids has been observed due to their antioxidant or prooxidant activity, depending on the concentrations. Reported in vitro and in vivo investigations suggest that the activation of the Nrf2/ARE pathway by either endogenous or exogenous stimuli under normal physiological conditions contributes to redox homeostasis, which may provide a mechanism for cancer chemoprevention. However, some flavonoids, such as luteolin, apigenin, myricetin, quercetin, naringenin, epicatechin, genistein, and daidzein, at low concentrations (1.5 to 20 µM) facilitate cancer cell growth and proliferation in vitro. Paradoxically, some flavonoids, including luteolin, apigenin, and chrysin, inhibit the Nrf2/ARE pathway in vitro. Therefore, even though flavonoids play a major role in cancer chemoprevention, due to their possible inducement of cancer cell growth, the effects of dietary flavonoids on cancer pathophysiology in patients or appropriate experimental animal models should be investigated systematically.

## 1. Introduction

### 1.1. Oxidative Stress and Antioxidant Defense System in Relation to Cancer

Several biological reactions that occur in the human body generate reactive oxygen species (ROS) [1]. Excessive generation of ROS can lead to oxidative stress that can potentially cause over 40 non-communicable diseases, such as certain cancers, diabetes mellitus, neurodegenerative diseases, and accelerated aging [2]. In addition to the primary endogenous ROS generation by many mechanisms, including the mitochondrial electron transport chain [1], exogenous stimuli, such as air pollutants, cigarette smoke, ionization radiation, xenobiotics, atmospheric pressure plasmas, and hypoxia, could induce ROS generation [3,4,5,6]. Non-communicable diseases and rapid aging induced by oxidative stress are mainly due to unrecoverable damages that occur to biological macromolecules, such as nucleic acids, proteins, and membranes [3,7]. For instance, DNA can be damaged in the forms of single-strand or double-strand DNA breakage, and stable modifications in the nitrogen bases of the pentose-phosphate backbone of DNA due to the ROS-induced oxidative stress [8,9]. If these damages are not repaired, they could lead to epigenetic alterations in proto-oncogenes and tumor suppressor genes, somatic gene mutations, and genomic instability, which could initiate carcinogenesis [8,10,11,12]. Therefore, it is essential to maintain the ROS at the non-deleterious basal level in cells, also called redox homeostasis, to prevent oxidative stress-induced DNA damage and the initiation of carcinogenesis, leading to neoplastic diseases [3,13].

The cellular antioxidant defense system is the primary mechanism to protect biological macromolecules from oxidative stress [3]. The enzymatic and non-enzymatic antioxidants of the antioxidant defense system are capable of neutralizing ROS, such as superoxide anion radical, hydrogen peroxide, and hydroxyl radical, and the secondary reactive species such as peroxyl and alkoxyl radicals, generated by their further oxidation [3,14,15]. In a cellular environment, superoxide anion radical is generated mainly due to the activities of lipoxygenase, nicotine adenine dinucleotide phosphate (NADPH) oxidase, cyclooxygenase, cytochrome P450, and xanthine oxidase [16,17]. This free radical is converted to hydrogen peroxide by superoxide dismutase (SOD) [18]. Hydrogen peroxide can also be produced by NADPH oxidase [19], xanthine oxidase [20] and amino acid oxidase enzymes [21], or as a result of oxygen consumption in the metabolic reactions that happen in peroxisome [22]. Hydrogen peroxides are further converted into water and oxygen by catalase (CAT) and glutathione peroxidase (GPx). GPx needs secondary enzymes such as glutathione reductase (GR) and co-factors, reduced glutathione and NADPH, to catalyze the conversion of hydrogen peroxide into water [18,23]. If hydrogen peroxides are not neutralized by CAT and GPx enzymes, hydrogen peroxides can react with superoxide radical, or undergo Fenton reactions or Haber–Weiss reactions in the presence of metal ions, such as copper and ferrous, to generate hydroxyl radical [24,25]. Hydroxyl radicals cause severe oxidative damage to DNA, and the inefficient or mis-repair of DNA can promote DNA mutations and genomic instability that can initiate carcinogenesis [3,13]. Therefore, it is essential to eliminate ROS to prevent oxidative stress-induced DNA damage. As such, pathways that regulate the expression of proteins related to the antioxidant defense system and other cytoprotective genes are vital in managing oxidative stress-induced DNA damage, and thus play a role in the prevention of cancer by limiting genomic instability [14,26].

In addition to oxidative stress-mediated cancer initiation, ROS play several roles in cancer therapy [27]. The ROS production in a cancer cell is higher than in a normal cell due to its hypermetabolism [28]. However, in cancer cells, oxidative balance is achieved due to their marked antioxidant capacity, facilitated by the activation of the antioxidant defense system and mediated by pathways such as Nrf2/ARE [27]. In anticancer therapy, oxidative stress-mediated damages to cancer cells are achieved by the induction of accelerated ROS generation and the inhibition of antioxidant defense systems, mainly through exogenous stimuli such as chemotherapeutic drugs, i.e., cisplatin, doxorubicin, and 2-methoxyestradiol [27,29,30]. ROS-mediated anticancer therapies are based on the generation of excessive ROS over the cytotoxic limit, which disrupts the redox homeostasis that kills cancer cells [31].

### 1.2. Mechanisms of Activation of the Antioxidant Defense System and Other Cytoprotective Genes

Activation of the antioxidant defense system and other cytoprotective genes, such as phase 2 detoxification enzymes, is mainly due to the activation of the nuclear factor erythroid 2-related factor 2 (Nrf2)/antioxidant response element (ARE) pathway in a cellular environment upon oxidative stress [32,33]. Briefly, the activation of this pathway is initiated by a transcription factor, Nrf2, which binds to the promoter region of the ARE, leading to the transcription of genes of antioxidant defense enzymes and phase 2 detoxifying enzymes. Thereby, these proteins restore the redox homeostasis by managing oxidative stress [34,35,36,37,38].

For the initiation of this pathway, Nrf2, a basic leucine-zipper transcription factor, needs to be activated. Under normal cellular physiological conditions, Nrf2 is bound to Kelch-like ECH-associated protein 1 (Keap1), which is an endogenous inhibitor of Nrf2, bound to actin fibers [39,40,41]. Interactions between Keap1 and Nrf2 via its motifs (Neh2 ETGE and DLG) lead to the activation of the Nrf2 ubiquitination process, which is mediated by the Cullin 3 (Cul3)-based E3 ligase complex [42]. The degradation of Nrf2 is rapidly undertaken in the 26S proteasome, leading to low levels of Nrf2 in the cytoplasm. This avoids the stabilization, phosphorylation and nuclear translocation of Nrf2, resulting in 15–40 min of Nrf2 half-life time depending on the type of cell [40,43,44,45].

However, oxidative stress or the presence of electrophilic compounds induce the activation of the Nrf2 pathway through canonical mechanisms. Herein, the cysteine residues (Cys151, Cys257, Cys273, Cys288, and Cys297) in Keap1 undergo conformational changes upon oxidation or alkylation, and dissociate Nrf2 from Keap1 [46,47,48]. In addition, the non-canonical activation of Nrf2 by the influence of proteins such as p62, p21, dipeptidyl peptidase III (DPP3), Wilms tumor gene on X chromosome (WTX), breast cancer gene 1 (BRCA1), and partner and localizer of BRCA2 (PALB2) also leads to the cytoplasmic stabilization of Nrf2 [48]. These proteins disrupt the direct interactions of Keap1 with Nrf2, by binding to either Keap1 or Nrf2. Collectively, the canonical and non-canonical activation of Nrf2 results in a reduction of Nrf2 ubiquitination and, consequently, its degradation [48]. The detached Nrf2 can be negatively regulated by several other proteins, such as glycogen synthase kinase 3 beta (GSK-3β). Herein, GSK-3β-mediated phosphorylation of specific serine residues, such as Ser335 and Ser338 (numbers in mouse sequence), in the Neh6 domain of Nrf2 creates a degradation domain, which can be recognized by the ubiquitin ligase adapter E3 ubiquitin–protein ligase. Thereafter, the proteasomal degradation of Nrf2 is facilitated via the Cul3-based E3 ligase complex [41]. However, the phosphorylation of serine 558 residue is located in the canonical nuclear export signal of the Nrf2 protein by 5′ adenosine monophosphate (AMP)-activated protein kinase (AMPK), which leads to the improved stability of the Nrf2 protein facilitating the nuclear translocation [49,50]. Once Nrf2 is translocated into the nucleus, it begins heterodimerization with another transcription factor called musculoaponeurotic fibrosarcoma (sMaf) [44,45]. The resulting complex binds to ARE and initiates the transcription of downstream genes belonging to the antioxidant defense system and phase 2 detoxifying enzymes. Once these proteins are expressed, functions such as the oxidizing of xenobiotics or drugs, the conjugation of oxidized metabolites, and the transportation of final metabolites out of the intracellular environment will ensure cytoprotection by restoring redox homeostasis [33]. Therefore, the identification of endogenous and exogenous molecules that can activate the Nrf2/ARE pathway offers potential protection against oxidative stress-mediated diseases.

Among many other phytochemicals, flavonoids have shown the potential to activate the Nrf2/ARE pathway in the absence of oxidative inducers [38,51]. However, the activation of the Nrf2/ARE pathway has not always been beneficial because the same flavonoids may promote the growth of cancer cells as well [52]. In normal cells, chemoprevention can be achieved by expressing Nrf2/ARE-driven cytoprotective genes [34,35,37,53,54,55]. However, a study conducted by Harris and colleagues in 2015 showed that the biosynthesis of cytoprotective molecules glutathione (GSH) and thioredoxin synergistically facilitates cancer initiation and progression in genetically engineered mice of mammary tumor initiation [56]. In cancer cells, the constitutive activation of the Nrf2/ARE pathway promotes cell growth [57], cell survival [58], continuous proliferation, and the renewal of stem cells of several types of cancers, as well as resistance to chemotherapies [33]. Some cancer chemotherapeutics are designed to inhibit the Nrf2/ARE pathway in cancer cells [59]. Therefore, certain flavonoids may have the potential for use in cancer treatment [60,61,62,63]. Thus, the remainder of this review focuses on understanding the dual role of flavonoids in cancer chemoprevention and the effects on cancer cell growth or cancer promotion with respect to the regulation of the Nrf2/ARE pathway.

### 1.3. Hormetic Effects of Dietary Flavonoids

The hormetic behavior of phytochemicals should be taken into consideration when determining the safe and effective concentrations for cancer prevention or cancer treatment. Hormesis is the bi-phasic concentration/dose–response, often depicted as the U-shaped dose–response curve of some dietary antioxidants, drugs, and toxins [64]. Accordingly, at low concentrations/doses, biologically active molecules such as flavonoids exert beneficial effects, such as stimulation for either adaptation or protection from a stress factor [64,65,66,67]. At high concentrations/doses, they may exert either detrimental/inhibitory or toxic effects on the cells or tissue microenvironment [64,66,67,68]. As such, hormetic compounds act as antioxidants at low doses and prooxidants at high doses [13,40,69,70,71]. For example, apigenin and luteolin at low concentrations (6.25 µM) exert stimulatory effects on the Nrf2/ARE pathway in human hepatocellular carcinoma HepG2 cells, significantly increasing the mRNA and protein expression of Nrf2 and heme oxygenase 1 (HO-1) with the activation of phosphatidylinositol-3-kinase (PI3K)/protein kinase B (Akt) and ERK1/2 signaling [65]. However, at high concentrations (50 and 100 µM), apigenin reduces the mRNA and protein levels of Nrf2, CAT activity, and intracellular glutathione levels in HepG2 cells [68]. Similarly, at low concentrations (10 µM), luteolin increases the GSH protein expression in human epithelial colorectal adenocarcinoma Caco-2 cells, and higher concentrations (above 15 µM) of luteolin decrease GSH expression, showing the hormetic effects [72]. Therefore, when considering flavonoids in cancer treatment and developing experiments to evaluate the effectiveness, their hormetic effect should be taken into consideration.

## 2. Role of the Nrf2/ARE Pathway in Cancer Chemoprevention

Activation of the Nrf2/ARE pathway in normal cells has been shown to have cancer chemopreventive effects on non-malignant cells under normal physiological conditions [33,40,73]. This can be mainly achieved by controlling redox homeostasis [74], which leads to genomic stability and cell survival that is facilitated by the activities of antioxidant defense enzymes (SOD, catalase, GPx, GSH synthase, glutathione S-transferase, thioredoxin, and GSH reductase) and phase 2 and 3 detoxifying enzymes (HO-1 and NAD(P)H quinone dehydrogenase 1 (NQO1), aldo-keto reductase, multidrug resistance-associated proteins, P-glycoprotein, organic anion-transporting polypeptide, ATP-binding cassette, heat shock proteins, glycation defense enzymes, and ferritin) [12,33,37,53,54]. These expressed proteins avoid oxidative stress-induced DNA damage by either reducing the exposure of DNA to carcinogens (exogenous or endogenous), inhibiting the activation of pro-carcinogens, or increasing the rate of detoxification of carcinogens [26,75,76]. Therefore, the inactivation of the Nrf2/ARE pathway could increase oxidative stress by generating ROS, create mutagenesis, and initiate carcinogenesis and tumor formation in normal cells [26,75,76]. For example, a decreased expression of the Nrf2 gene increases the risk of lung cancer among smokers [77]. Furthermore, decreased levels of phase 2 enzymes, such as HO-1 and Nrf2 proteins, in Nrf2-knockout animal models, such as female C57BL/6 mice, increases susceptibility towards 7,12-dimethylbenz(a)anthracene-induced skin tumorigenesis [78]. Therefore, many investigations propose the activation of the Nrf2/ARE signaling pathway as a potential cell signaling pathway in cancer chemoprevention [35].

### 2.1. Activators of Nrf2/ARE in Non-Cancer Experimental Models

Investigations into the activation of the Nrf2/ARE pathway have shown that some vitamins and a diverse range of dietary phytochemicals, including flavonoids, sulforaphanes, alkaloids and polyphenols, activate the pathway by different mechanisms in non-cancer experimental models [40] (Table 1). Further, several endogenous signaling molecules, such as protein kinase-like endoplasmic reticulum-resident kinase (PERK), c-Jun *n*-terminal kinase (JNK), extracellular signal-regulated protein kinase (ERK) and p38, are under normal conditions known to give similar results [79,80]. The activation of the Nrf2/ARE pathway mainly occurs through the disruption of Keap1 and Nrf2 interactions (either through canonical or non-canonical mechanisms) [48], Nrf2 phosphorylation [47], and the prevention of Nrf2 ubiquitination [81]. In addition, some of the activators facilitate Nrf2 nuclear translocation and the transcription of cytoprotective genes associated with ARE [81].

Resveratrol, a stilbene derivative, and RTA-408 (omaveloxolone), a synthetic terpenoid, activate the Nrf2 pathway through canonical mechanisms [47,82,83,84,85]. Canonical activators that obstruct the interaction of the Keap1/Nrf2 system possess electrophilic properties and react with the cysteine residues (i.e., Cys151, Cys257, Cys273, Cys288, and Cys297) of Keap1 via either oxidation or alkylation in order to dissociate Nrf2 from Keap1 [35,46,47,53]. For example, in a phase 3 clinical trial on chronic subclinical inflammation and redox status, 500 mg of resveratrol in one tablet/day up to 30 days (Table 1) has shown to be disruptive to the Nrf2–Keap1 interactions via conformational changes. These changes occurred due to electrophilic modifications in the Keap1-Cys151 thiol group [47,85]. The Cul3-based E3 ligase complex binds and interacts with Keap1 to facilitate Nrf2 polyubiquitination, which promotes Nrf2 degradation at the 26S proteasome. Therefore, post-translational modifications in Cys151 lead to the dissociation of the Cul3-based E3 ligase complex from Keap1 and Nrf2 stabilization [43,44,45,47,82]. Thereby, it prevents Nrf2 proteasomal degradation by ubiquitination, and facilitates ARE-mediated gene expression [81,86,87]. Similar results were observed with RTA-408 in a clinical trial on inflammation and pain due to ocular surgery (1% ophthalmic suspension of RTA-408 twice a day for 14 days) [47,83].

Sequestosome-1, an endogenous signaling molecule, activates the Nrf2 pathway via non-canonical mechanisms by blocking Nrf2 binding to Keap1 [88] (Table 1). Sequestosome 1, also called p62, not only competes with Nrf2 to bind to Keap1 and block the formation of Nrf2–Keap1 complex, but also promotes the autophagic degradation of Keap1 [48,88]. For example, Nrf2 silencing downregulates p62 expression while upregulating Keap1 expression at the mRNA and protein levels in vascular smooth muscle cells. Conversely, p62 silencing dramatically upregulates Keap1 and downregulates Nrf2 at the mRNA and protein levels, suggesting p62 may be effective in downregulating Keap1 protein via autophageal degradation [88].

Most of the endogenous activators of the Nrf2/ARE pathway act by stimulating the phosphorylation of Nrf2, which leads to the detachment of Nrf2 from Keap1 [53]. For example, the PERK-mediated direct phosphorylation of Nrf2 in mouse embryonic fibroblasts results in the dissociation of Nrf2 from Keap1 (Table 1) [79]. Similarly, JNK 1 and 2, ERK2 and p38 phosphorylate Nrf2 at the serine (Ser212, Ser400, Ser558, Ser577) and threonine (Thre559) residues in human embryonic kidney HEK 293T cells [80]. It is also suggested that the above-mentioned endogenous activators of the Nrf2/ARE pathway can be activated by phytochemicals such as diallyl sulfide. Diallyl sulfide phosphorylates ERK and p38 in human embryonic lung MRC-5 cells, and facilitates the dissociation of Nrf2 from Keap1 and nuclear translocation [89].

Most of the endogenous activators of Nrf2 are protein kinases, which seem to facilitate the nuclear translocation of phosphorylated Nrf2 [49,90,91]. Nrf2 phosphorylation mediated by AMPK, casein kinase 2, PERK, ERK and p38 facilitates Nrf2 nuclear translocation [90]. PI3K signaling is also involved in the activation of the Nrf2/ARE pathway through its downstream regulator Akt, which facilitates Nrf2 nuclear translocation and the following ARE gene transactivation [92]. Therefore, the influence of endogenous signaling molecules, phytochemicals and synthetic chemicals upon activation of Nrf2/ARE at different stages of the pathway will be vital in exerting chemopreventive effects upon the activation of the Nrf2/ARE pathway [40,47,79,80].

### 2.2. Flavonoids: Nrf2/ARE Activation in Non-Cancer Experimental Models

**Figure 1:**
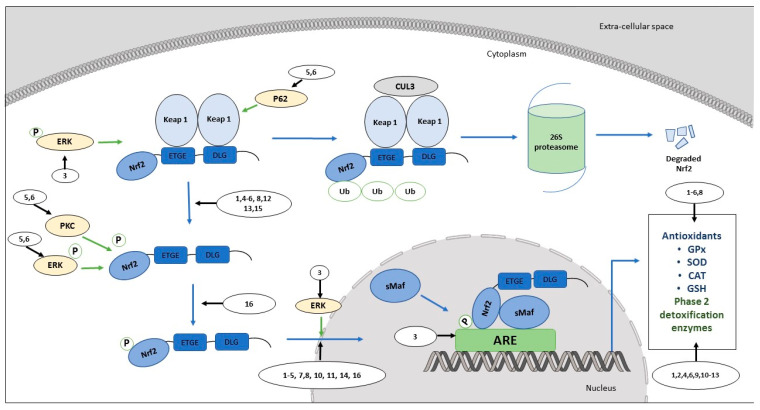
Role of dietary flavonoids in the regulation of the Nrf2/ARE pathway in normal cells.

Flavonoids are among the most noticeable dietary phytochemicals that activate the Nrf2/ARE pathway under normal physiological and induced conditions (Table 1) (Figure 1). The sub-class flavones (luteolin, baicalin and apigenin) are more prominent in upregulating Nrf2 protein expression in both in vitro and pre-clinical studies [93,94,95]. For example, luteolin upregulates Nrf2 protein expression in relieving high glucose-induced cell injury in rat myoblast H9C2 cells at rat-physiological concentrations [93,96]. Baicalin also increases Nrf2 protein expression against hypoxia-induced apoptosis in rat myoblast H9C2 cells, although the required concentration is higher than rat-physiological concentrations [97,98]. Furthermore, the intraperitoneal administration of baicalin demonstrates a similar effect in male Sprague-Dawley rats after inducing subarachnoid hemorrhage by endovascular perforation [94,99]. A study conducted by Xu and colleagues showed that apigenin increases the Nrf2 protein level as a response to *tert*-butyl hydroperoxide (t-BHP)-induced oxidative cell injury in human retinal pigment epithelial ARPE-19 cells, at much higher concentrations compared to other flavones [95]. Similarly, apigenin upregulated Nrf2 expression at transcriptional and translational levels, at much higher concentrations, against hydrogen peroxide-induced oxidative stress and cell injury in human renal tubular epithelial HK-2 cells [100]. However, the tested concentrations in both ARPE-19 and HK-2 cell lines were much higher than the bioavailable apigenin levels in human plasma [95,100,101].

Further, the upregulation of Nrf2 protein was observed in flavonols (quercetin), flavanones (naringenin, hesperidin), and flavan-3-ols (epigallocatechin-3-gallate) [102,103,104,105]. Naringenin, a flavanone found in citrus fruits, upregulates the Nrf2 protein in hypoxia-induced neuron cells derived from neonatal Sprague-Dawley rats [103]. However, this was at comparatively higher concentrations that may not be achievable in pre-clinical studies, considering the limited bioavailability of naringenin in rats [103,106,107]. Quercetin upregulates Nrf2 protein expression in different human cell lines, such as human skin keratinocytes HaCaT, BJ foreskin fibroblast, and human umbilical vein endothelial cells (HUVECs), but at concentrations higher than physiologically relevant concentrations considering the low bioavailability of quercetin in the human diet [102,108,109,110]. Furthermore, the oral administration of quercetin upregulates Nrf2 protein levels against lipopolysaccharide (LPS)-induced intestinal oxidative stress in broiler chicken [111]. Furthermore, pre-clinical studies on the oral administration of naringenin show the upregulation of the Nrf2 protein in male C57BL/6 mice with 6-hydroxydopamine (6-OHDA)-induced neurotoxicity at a concentration which is not toxic [112,113]. Furthermore, both hesperidin (oral) and epigallocatechin-3-gallate (intraperitoneal) have shown that similar upregulations can be achievable at non-toxic concentrations in male Sprague-Dawley rats with methotrexate (MTX)-induced hepatotoxicity and testicular ischemia-induced oxidative stress, respectively [104,105,114,115]. In contrast, mRNA levels of Nrf2 were upregulated only in flavones such as baicalin (chicken with *Mycoplasma gallisepticum* infection-induced oxidative stress) and apigenin (human retinal pigment epithelial ARPE-19 cells with *t*-BHP-induced oxidative cell injury) in pre-clinical and in vitro models [95,116]. However, the molecular mechanisms of the flavonoid-mediated increase of cellular Nrf2 mRNA and protein levels remain unclear.

The activation of Nrf2 is necessary for the progression of the pathway either by canonical or non-canonical mechanisms [46,47,48]. Baicalin and its aglycon baicalein activate the Nrf2/ARE pathway in acetaminophen-induced L-02 (synonym: HL-7702) human liver cells through non-canonical activation of Nrf2 via p62 [117]. Baicalin and baicalein increase the expression of the p62 protein in L-02 cells, which competes with Nrf2 for binding to the Nrf2 binding site in the Keap1-kelch domain [117]. The inductive effects of baicalin and baicalein in L-02 cells further continues as baicalin, and baicalein itself, compete with Nrf2 to bind to the Nrf2 binding site [117]. Hydroxyl groups at C5 and C6 of the A-ring of these two flavones might be useful in anchoring them to the Nrf2 binding site in the Keap1-kelch domain through hydrogen bond formation [118]. Furthermore, baicalin and baicalein phosphorylate ERK1/2 and protein kinase C (PKC). Thereby, phosphorylated ERK1/2 and PKC phosphorylate Nrf2. Therefore, these flavones increase Nrf2 stabilization by preventing Nrf2 ubiquitination [117]. However, the inductive effects of effective baicalin and baicalein concentrations in L-02 cells are higher than the physiological (intraperitoneally administered) concentrations of these two flavones in human primates and monkeys [117,119,120].

The nuclear translocation of phosphorylated Nrf2 is a necessity in order to proceed with ARE-driven gene transcription. [49,50]. Many subclasses of flavonoids, including flavones (luteolin, baicalein, chrysin, and apigenin), flavonols (myricetin and quercetin), flavanones (eriodyctiol), flavan-3-ols (epicatechin), isoflavones (genistein), anthocyanidins (cyanidin-3-*O*-glucoside; C3G) and chalcones (butein), promote Nrf2 nuclear translocation (Table 1). Luteolin and epicatechin upregulate the Nrf2 nuclear translocation in mice cells; mouse testis Sertoli TM4 (triptolide-induced apoptosis) and hemoglobin toxicity induce the primary astrocytes of mice, respectively [121,122]. Similarly, chrysin and quercetin upregulate Nrf2 nuclear translocation in rat hepatocytes (*t*-BHP-induced oxidative stress) and rat intestinal epithelial IEC-6 cells [123,124]. Furthermore, in rat hepatocytes, the chrysin-mediated upregulation of the phosphorylated ERK1 increases Nrf2 nuclear translocation [124]. Therefore, ERK1-mediated influences may be due to the improved stability of Nrf2 upon Nrf2 phosphorylation, which prevents Nrf2 ubiquitination and degradation [117]. However, the tested concentrations of luteolin (mouse testis Sertoli TM4 cells), epicatechin (primary astrocytes from mice), quercetin (rat hepatocytes) and chrysin (IEC-6 cells) in the above murine cells are higher than the achievable physiological concentrations in murine models upon oral administration [51,125,126,127]. Baicalein upregulates the Nrf2 nuclear translocation against high glucose-induced oxidative stress in L-02 liver cells [119,120]. Furthermore, C3G upregulates Nrf2 nuclear translocation in HUVECs challenged with tumor necrosis factor-α [128]. However, the tested concentrations of C3G on HUVECs are much higher than the serum levels that can be achieved in humans upon oral uptake [129]. Apigenin also facilitates nuclear translocation in human retinal epithelial ARPE-19 cells at concentrations higher than those physiologically available in humans [95,101]. Similarly, butein upregulates Nrf2 nuclear translocation against hydrogen peroxide-induced oxidative stress in human dental pulp cells [55,130]. Further, due to the lack of availability of clinical data on the bioavailability of butein, the physiological relevance of tested concentrations of butein is mostly unknown.

Furthermore, promising results on the upregulation of Nrf2 nuclear translocation were observed in several pre-clinical studies (ICR mice, Sprague-Dawley rats, Kunming mice, and broiler chicken) [51,111,120,131,132]. Luteolin, baicalein, myricetin, quercetin and genistein demonstrate their ability in upregulating Nrf2 nuclear translocation [51,111,120,131,132]. Luteolin increases Nrf2 nuclear translocation in male Sprague-Dawley rats with intracerebral hemorrhage-induced secondary brain damage (intraperitoneal administration) and male ICR mice (oral administration) at concentrations which are not toxic [51,131,133,134,135]. Similarly, the oral administration of baicalein facilitates Nrf2 nuclear translocation in male type 2 diabetes mellitus (T2DM) Kunming mice with high glucose-induced oxidative stress, at a concentration much lower than the maximum tolerable levels for mice [120,136]. Further, myricetin (oral administration) was effective in upregulating Nrf2 nuclear translocation against cuprizone-induced demyelination in male Kunming mice [132]. The concentrations of myricetin tested on Kunming mice are much lower than the sub-lethal concentrations of myricetin for mice [132,137]. Furthermore, both quercetin (oral administration) and genistein (intraperitoneal administration) upregulate Nrf2 nuclear translocation in broiler chickens (LPS-induced intestinal oxidative stress) and male Sprague-Dawley rats (cerebral ischemia-induced oxidative stress) [111,138]. Further, the tested concentrations of genistein on Sprague-Dawley rats were much lower than concentrations that show toxic effects in mice [139].

The downstream activation of the Nrf2/ARE pathway upon Nrf2 nuclear translocation by flavonoids has been demonstrated. The overexpression of antioxidant defense genes (GSH, SOD, GPx and CAT) and phase 2 detoxifying genes (HO-1 and NQO-1) was observed due to flavones (luteolin, apigenin, baicalin, baicalein, chrysin), flavanones (naringenin, and hesperidin), flavonols (quercetin, rutin), anthocyanins (C3G) and isoflavones (genistein) (Table 1). Baicalein upregulates the expression of downstream target genes, SOD, CAT, and GSH against high glucose-induced oxidative stress in L-02 liver cells at physiologically higher concentrations [119,120].

Chrysin upregulates cellular GSH proteins (antioxidant defense gene) in relieving the *t*-BHP-induced oxidative stress of rat hepatocytes [124]. Baicalin and naringenin upregulate phase 2 detoxifying enzymes at the protein level against hypoxia-induced oxidative stress or apoptosis in H9C2 (HO-1) and Sprague-Dawley neuronal cells (HO-1 and NQO-1), respectively [97,103]. Although the concentrations tested were higher than the physiologically relevant range, rutin and C3G upregulate the expressions of HO-1 and NQO-1 in the mRNA levels of HaCaT and HUVECs, respectively [110,128,129,140]. Further, apigenin enhances the activity of SOD, CAT, and GPx in human retinal epithelial ARPE-19 cells, and the expression of HO-1 in the mRNA levels of human renal tubular epithelial HK-2 cells, but at much higher concentrations than can be achieved physiologically in humans [95,100,101].

In preclinical studies, the oral administration of baicalein and quercetin upregulates proteins related to antioxidant defense genes against relieving oxidative stress in high glucose-induced male T2DM Kunming mice (SOD, CAT, and GSH) and LPS-induced broiler chicken (SOD and CAT), respectively [111,120]. In contrast, the upregulation of both antioxidant defense (SOD and GSH) and phase 2 detoxifying genes (NQO-1 and mRNA HO-1) was observed upon intraperitoneal administration of baicalin in male Sprague-Dawley rats after inducing subarachnoid hemorrhage [94]. Luteolin upregulates phase 2 detoxifying enzymes (HO-1 and NQO-1) in male Sprague-Dawley rats with intracerebral hemorrhage-induced secondary brain damage (intraperitoneal administration) and male ICR mice (oral administration) [51,131]. In contrast, the upregulation of HO-1 was observed at the protein level against cerebral ischemia-induced oxidative stress in Sprague-Dawley rats upon intraperitoneal administration of genistein [138]. Furthermore, the oral administration of baicalin and hesperidin upregulated HO-1 at both the protein and mRNA levels against *Mycoplasma gallisepticum* infection-induced oxidative stress in chicken and MTX-induced hepatotoxicity in male Sprague-Dawley rats, respectively [104,116]. More importantly, the above upregulations of either or both antioxidant and phase 2 detoxifying enzymes by luteolin (ICR mice and Sprague-Dawley rats), baicalein (T2DM Kunming mice), baicalin (Sprague-Dawley rats), hesperidin (Sprague-Dawley rats) and genistein (Sprague-Dawley rats) were observed in concentrations lower than toxic or lethal in in vivo studies [99,114,133,134,136,139]. Based on the reported literature, further investigations should be carried out so as to better understand the molecular mechanisms of the effects of flavonoids in facilitating the activation, stabilization and nuclear translocation of Nrf2, and ARE-driven gene expression.

**Table 1:** Activators of the Nrf2/ARE pathway in non-cancer experimental models: phytochemicals and other signal molecules.

Group	Compound	Effective Concentration	Experimental Model	Mode of Action	Reference
**Phytochemicals–Polyphenols**					
Phenolic acid	Ellagic acid	25–50 µM	Human keratinocyte HaCaT cells	↑ Nrf2 nuclear translocation. ↑ SOD enzyme activity.	[141]
	Chlorogenic acid	100 µM	Human retinal pigment epithelial ARPE-19 cells	↑ mRNA expression of Nrf2 and SOD.	[142]
		500 mg/kg of body weight orally	Sprague-Dawley rats	↑ mRNA expression of Nrf2. ↑ SOD and GSH activities.	[143]
Proanthocyanidin	Procyanidin C1	5–10 µM	Mouse hippocampal neuronal HT22 cells	↑ Nrf2 nuclear translocation. ↑ HO-1 protein.	[144]
Lignans	Sesamin	100 mg/kg body weight intraperitoneally	C57BL/6 mice	↑ SOD and CAT activities. ↑ GSH and Nrf2 protein.	[145]
		10 µM	Primary chondrocytes	↑ Nrf2 and HO-1 proteins.	[146]
Coumarins	Fraxin	50 mg/kg of body weight up to 5 days orally	Sprague-Dawley rats	↑ cellular GSH levels.	[147]
Stilbene derivative	Resveratrol	5 µM	Primary human coronary artery endothelial cells	↑ mRNA expression of NQO1.	[148]
		500 mg in a tablet/day up to 30 days in the morning fasting	Phase 3 clinical trial on chronic subclinical inflammation and redox status	↑ electrophilic modification of Keap1-Cys-151	[47,85]
		215 mg in a tablet/day up to 52 weeks	Phase 2 clinical trials on Alzheimer’s disease	↑ electrophilic modification of Keap1-Cys-151	[47,149]
Curcumminoid	Curcumin	5 µM	Human extravillous trophoblast HTR8/Sveo cells	↑ CAT and GSH activities.	[150]
		400 mg/kg body weight/day orally up to 21 days	White Pekin ducklings	↑ CAT, SOD, and GPx activities.	[151]
		800 mg/day in two capsules up to 7 days	Phase 3 clinical on diabetic nephropathy	↑ electrophilic modifications of Keap1-Cys-151	[47,82]
		15 µM	Human retinal pigment epithelial ARPE-19 cells	↑ Nrf2 protein. ↑ HO-1 activity.	[152]
		15–30 µM	Porcine renal epithelial proximal tubule LLC PK1 cells	↑ ARE binding activity. ↑ Nrf2 protein. ↑ HO-1 activity.	[153]
		10 µM	Rat kidney epithelial NRK-52E cells	↑ ARE binding activity.	[153]
		200 mg/kg body weight twice a week for 6 weeks	Kunming (KM) mice	↑ Nrf2 nuclear translocation. ↑ HO-1 and NQO-1 proteins.	[154]
**Phytochemicals–Polyphenols–Flavonoids**					
Flavone	Luteolin	0.1 mg/kg body weight/day for 7 days at two time points orally.	ICR mice	↑ Nrf2 nuclear translocation. ↑ HO-1 and NQO-1 proteins.	[51]
		10 mg/kg body weight intracerebrally injected	Sprague-Dawley rats	↑ Nrf2 nuclear translocation. ↑ HO-1 and NQO-1 proteins.	[131]
		5–10 µM	Rat myoblast H9c2 cells	↑ Nrf2 protein. ↑ mRNA expression of SOD, NQO-1 and HO-1.	[93]
		5 µM	Mouse testis sertoli TM4 cells	↑ Nrf2 nuclear translocation.	[121]
	3,5-di-O-Methyl Gossypetin	10–25 µg/mL	Human keratinocyte HaCaT cells	↑ Nrf2 nuclear translocation. ↑ GSH, SOD and HO-1 proteins.	[55]
	Baicalein	160 mg/kg/day for 8 weeks orally	T2DM Kunming mice	↑ Nrf2 nuclear translocation. ↑ SOD, CAT, GSH proteins.	[120]
		50 µM	Human liver L-02 cells	↑ p62 protein. ↑ Nrf2 dissociation from Keap1. ↑ Nrf2 phosphorylation via phosphorylation of ERK1/2 and protein kinase C.	[117]
		20 µM	Human liver HL-7702 cells	↑ Nrf2 nuclear translocation. ↑ SOD, CAT, GSH protein.	[120]
	Baicalin	50 mg/kg body weight twice after 2 and 12 h of subarachnoid hemorrhage intraperitoneally.	Sprague-Dawley rats	↑ SOD, GSH, NQO-1, and Nrf2 proteins. ↑ mRNA expression of HO-1.	[94]
		75 µM	Rat myoblast H9C2 cells	↑ Nrf2 and HO-1 proteins.	[97]
		450 mg/kg body weight/day up to 7 days orally.	Chicken	↑ Nrf2 and HO-1 proteins. ↑ mRNA expression of Nrf2 and HO-1.	[116]
		50 µM	Human liver L-02 cells	↑ p62 protein. ↑ Nrf2 dissociation from Keap1. ↑ Nrf2 phosphorylation via phosphorylation of ERK1/2 and protein kinase C.	[117]
	Chrysin	10–25 µM	Rat hepatocytes	↑ Nrf2 nuclear translocation via ERK2 signaling. ↑cellular GSH protein. ↑ ARE binding ability.	[124]
	Apigenin	400 µM	Human retinal pigment epithelial ARPE-19 cells	↑ mRNA expression of Nrf2. ↑ Nrf2 protein. ↑ Nrf2 nuclear translocation. ↑SOD, CAT, and GPx activities.	[95]
		200 µM	Human renal tubular epithelial HK-2 cells	↑ mRNA expression of Nrf2 and HO-1. ↑ Nrf2 protein.	[100]
Flavonol	Myricetin	100 mg/kg/day for 6 weeks orally	Kungming mice	↑ Nrf2 nuclear translocation.	[132]
	Quercetin	30 µM	Human keratinocyte HaCaT and BJ foreskin fibroblast cells	↑ Nrf2 protein.	[102]
		200 mg/kg body weight/day for 20 days orally	Broiler chicken	↑ Nrf2 protein. ↑ Nrf2 nuclear translocation. ↑ SOD and CAT proteins.	[111]
		100 µM	Intestinal epithelial IEC-6 cells	↑ Nrf2 nuclear translocation.	[155]
		10 µM	Human umbilical endothelial cells	↑ Nrf2 protein.	[108]
	Rutin	44 µM	Human keratinocyte HaCaT cells	↑ mRNA expression of HO-1 and NQO-1.	[140]
Flavanone	Naringenin	80 µM	Sprague-Dawley rat neuron cells	↑ Nrf2, HO-1, and NQO-1 proteins.	[103]
		70 mg/kg body weight/day up to 4 days orally	C57BL/6 mice	↑ Nrf2 protein.	[112]
	Hesperidin	50 mg/kg body weight for 28 days orally	Sprague-Dawley rat	↑ Nrf2 and HO-1 proteins. ↑ mRNA expression of HO-1.	[104]
Flavan-3-ol	Epicatechin	10–100 µM	Primary astrocytes from WT and Nrf2 deficient KO mice	↑ Nrf2 nuclear translocation.	[122]
	Epigallocatechin-3-gallate (EGCG)	40 mg/kg body weight/day for 3 days intraperitoneally or a single dose of 50 mg/kg body weight intraperitoneally	Sprague-Dawley rat	↑ Nrf2 protein.	[105,156]
Isoflavones	Genistein	1 mg/kg body weight intraperitoneally	Sprague-Dawley rat	↑ Nrf2 nuclear translocation. ↑ HO-1 protein.	[138]
Anthocyanin	Cyanidin-3-*O*-glucoside (C3G)	20–40 µM	Human umbilical vein epithelial cells	↑ Nrf2 nuclear translocation. ↑mRNA expression of NQO-1 and HO-1.	[128]
Chalcone	Butein	20 µM	Human dental pulp cells	↑ Nrf2 nuclear translocation.	[130]
**Other Phytochemicals**					
Sulfur-containing	Sulforaphane	5 µM	Mouse skin JB6 P+ cells	↑ Nrf2 nuclear translocation. ↑ HO-1 and NQO-1 proteins.	[157]
	Diallyl sulfide	15 µM	Human embryonic lung MRC-5 cells	Dissociates Nrf2 from Keap1 through phosphorylated ERK and p38 interactions. ↑ Nrf2 nuclear translocation.	[89]
		150 mg/kg body weight/day intraperitoneally for 6 days	Wistar rats	↑ Nrf2 protein. ↑SOD, CAT, GPx, GR, GST, and quinone reductase activities.	[158]
Alkaloids	Berberine	200 mg/kg body weight/day orally for 16 weeks	Wistar rats	↑ mRNA expression of Nrf2.	[159]
**Vitamins**					
Fat-soluble vitamins	Vitamin D	40 000 U/kg/week of body weight intratracheally for 8 weeks	C57BL/6 mice	↑ mRNA expression Aldo-keto reductase family 1 member C1 (AKR1C1) and GCLM.	[160]
	Vitamin E	100 mg/kg body weight/day intraperitoneally for 6 days	Balb/c mice	↑ Nrf2 and HO-1 protein levels.	[161]
	Vitamin A	100,000 U/kg body weight/day subcutaneously for 14 days	Wistar rats	↑ Nrf2 nuclear translocation. ↑ HO-1 and NQO 1 proteins.	[162]
Water-soluble vitamins	Vitamin C	27–65 mg/kg body feed twice a day for 8 weeks	Juvenile *Sillago sihama*	↑ mRNA expression of Nrf2, CAT, SOD, GPx, GR, and GST in intestine and liver cells.	[163]
	Vitamin B2	30 mg/kg body weight/day intra-gastrically	APP/PS1 double transgenic mice	↑ SOD, CAT, GSH, and GPx activities. ↑ Nrf2 expression. ↓ Keap1 expression.	[164,165]
**Endogenous Signaling Molecules**					
Protein kinases	PI3K	N/A	Human retinal pigment epithelial RPE-19 cells	↑ Nrf2 nuclear translocation.	[92]
	JNK 1 & 2	N/A	Human embryonic kidney HEK 293T cells	Phosphorylates Nrf2 at S212, S408, S558, S577, and T559.	[80]
	p38	N/A	Human embryonic kidney HEK 293T cells	Phosphorylates Nrf2 at Ser212, Ser408, Ser558, Ser577, and Thre559.	[80]
	AMPK	N/A	Human embryonic kidney HEK 293T cells	Phosphorylates Nrf2 at the Ser558 residue. ↑ Nrf2 nuclear translocation.	[49]
	ERK2	N/A	Human embryonic kidney HEK 293T cells	Phosphorylates Nrf2 at Ser212, Ser408, Ser558, Ser577, and Thre559.	[80]
	Casein kinase 2	N/A	Human embryonic kidney HEK 293T cells	↑ Nrf2 phosphorylation. ↑ Nrf2 nuclear translocation.	[90]
	PKC	N/A	New Zealand white rabbits	↑ Nrf2 nuclear translocation.	[91]
	PERK	N/A	Mouse embryonic fibroblasts	Dissociates Nrf2 from Keap1 by phosphorylation of Nrf2.	[79]
Autophagy-substrate proteins	Sequestosome 1 (p62)	N/A	Human aortic smooth muscle cells	Competes with Nrf2 to bind with Keap1.	[88]
**Synthetic compounds**					
Synthetic triterpenoids	Bradoxolone-methyl (CDDO-Me)	25–50 mg/day for 52 weeks	Phase 2 clinical on diabetic nephropathy	↑ electrophilic modification of Keap1-Cys-151.	[47,84]
Synthetic triterpenoids	RTA-408 (omaveloxolone)	1% ophthalmic suspension for twice a day for 14 days	Phase 2 clinical trial on inflammation and pain following ocular surgery	↑ electrophilic modification of Keap1-Cys-151.	[47,83]
Synthetic lignans	LGM2605 (Secoisolariciresinol diglucoside)	50 µM	Murine peritoneal macrophages derived from C57BL/6J mice	↑ mRNA expression of GST and redoxin reductase 1.	[166]

Abbreviations—HaCaT: human skin keratinocytes; ARPE-19: human retinal pigment epithelial cell line; HT22: mouse hippocampal neuronal cell line; LLC PK_1_: porcine renal epithelial proximal tubule cell line; NRK-52E: rat kidney epithelial cell line; IEC-6: intestinal epithelial cell line; HK-2: human renal tubular epithelial cell line; HL-7702/ L-02: human liver cell line; MRC5: human embryonic lung cell line; HTR8/Sveo: extravillous trophoblast cell line; HEK 293T: human embryonic kidney 293T cell line; HT22: mouse neuronal cell line; H9C2: rat myoblast cell line: TM4: mouse Sertoli cell line; T2DM mice: type 2 diabetes mellitus mice; BJ: human foreskin fibroblast cell line; WT: wild type; KO: knock-out; JB6 P+: mouse skin cells; APP/PSI: ARTE1; GCLM: glutamate-cysteine ligase modifier; GSH: glutathione; CAT: catalase; SOD; superoxide dismutase; GPx: glutathione peroxidase; ARE: antioxidant response element; ERK: extracellular signal-regulated protein kinase; GSK-3β: glycogen synthase kinase 3; Akt: protein kinase B; GR: glutathione reductase; NQO-1: NAD(P)H quinone dehydrogenase 1; HO-1: heme oxygenase 1; GST- glutathione S-transferase; Ser212: serine residue 212; Ser408: serine residue; Ser558: serine residue 558; Ser577: serine residue 577; Thre559: threonine residue 559; PI3K: phosphorylation of phosphatidylinositol 3-kinase; JNK: N-terminal kinase; AMPK: 5′ adenosine monophosphate-activated protein kinase; PKC: protein kinase C; PERK: protein kinase-like endoplasmic reticulum-resident kinase; p62: sequestosome 1; Keap1: Kelch-like ECH-associated protein 1; Nrf2: Nuclear factor erythroid 2 p45 (NF-E2)-related factor; ↑ increase; ↓ decrease.

In normal cells, flavonoids have been shown to activate the Nrf2/ARE pathway in maintaining redox homeostasis. Under normal physiological conditions, Keap1 protein inhibits the activation of the Nrf2 protein by its interactions with the Nrf2 protein and ubiquitination-associated Nrf2 degradation. Upon oxidative stress caused by ROS, the oxidation of cysteine residues of Keap1 makes the Nrf2 dissociate from the Keap1 protein, followed by the stabilization of Nrf2 via phosphorylation. Phosphorylated Nrf2 translocates into the nucleus and binds to ARE along with the sMaf transcription factor. ARE-driven downstream antioxidant defenses and phase 2 detoxifying proteins will be expressed, leading to the restoration of normal physiological conditions via the detoxification of xenobiotics, drug transportation, and the neutralization of reactive species avoiding DNA damage and subsequent carcinogenesis. Dietary flavonoids activate the Nrf2/ARE pathway by influencing the pathway at different stages, and thus have potential effects on cancer chemoprevention.

1: Luteolin; 2: 3,5-di-O-Methyl Gossypetin; 3: Chrysin; 4: Apigenin; 5: Baicalein; 6: Baicalin; 7: Myricetin; 8: Quercetin; 9: Rutin; 10: Genistein; 11: C3G; 12: Naringenin; 13: Hesperidin; 14: Epicatechin; 15: EGCG; 16: Butein.

Keap1: Kelch-like ECH-associated protein 1; Nrf2: Nuclear factor erythroid 2 p45 (NF-E2)-related factor; sMaf: Small musculoaponeurotic fibrosarcoma protein; ARE: Antioxidant response element; GSH: glutathione; SOD: superoxide dismutase; CAT: Catalase; GPx: Glutathione peroxidase.

(The figure was adapted from Wu et al., 2019 [33])

## 3. Promotion of Cancer Cell Proliferation by Activation of Nrf2/ARE: Nrf2-Associated Cell Signaling and Mechanisms

The constitutive activation of Nrf2 promotes the development of different types of cancers as well as the resistance of cells to anti-cancer drugs [167]. The cellular mechanisms that over-activate the Nrf2/ARE pathway include disruption of interactions between Nrf2 and Keap1, the reduction of Keap1 protein expression, and the increase in Nrf2 protein expression [33]. The interactions between Nrf2 and Keap1 are inhibited by somatic mutations acquired in the Nrf2, CUL3 and/or Keap1 genes in cancer cells [168,169,170]. Furthermore, the Nrf2 protein can acquire mutations during protein translation by skipping exons of the Nrf2-coding mRNA strand [171]. The resultant Nrf2 or/and Keap1 mutants disrupt Nrf2 binding to Keap1 [33,169,170,171]. Similarly, the generated Keap1 and/or CUL3 mutants in cancer cells prevent CUL3–Keap1–Nrf2 complex formation, blocking Nrf2 ubiquitination [33,168,170]. Further, Nrf2 ubiquitination and the binding affinity of Nrf2 to Keap1 is reduced in cancer cells by the competition of endogenous signaling molecules, such as p62, partner and localizer of *BRCA2* (PALB2), and dipeptidyl-peptidase 3 (DPP3), with Nrf2 to bind to Keap1 [172,173,174,175,176]. Furthermore, the succination of cysteine molecules in Keap1 facilitates the dissociation of Nrf2 from Keap1 [177]. The reduction of Keap1 protein levels in cancer cells is mostly due to the epigenetic alteration of Keap1 through the hypermethylation of the CpG islands in the Keap1 promoter region [178], which thereby releases Nrf2 from the inhibitory regulation of Keap1 [178]. Further, the transcription of the Nrf2 protein is increased by either epigenetic changes in Nrf2, mutations on specific tumor suppressor genes (*PTEN*: Phosphatase and tensin homolog), or oncogenes (*Myc*, K-*Ras*, and B-*Raf*) [33,179,180]. However, the mechanism of the overactivation of Nrf2/ARE could be different from one cancer type to another [181]. As a result of the overactivation of the Nrf2/ARE pathway, cancer cells continue to grow and proliferate continuously, evading cellular apoptotic signals and promoting the self-renewal capacity of cancer stem cells [33].

**Figure 2:**
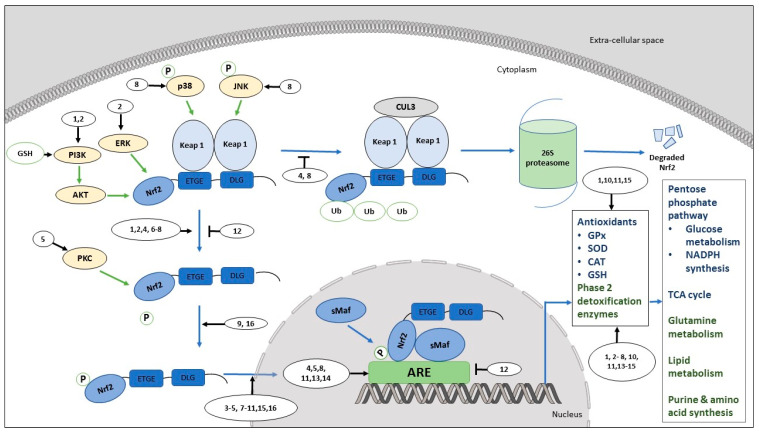
Role of dietary flavonoids in the Nrf2/ARE pathway in cancer cells.

The promotion of cancer cell proliferation by over activation of the Nrf2/ARE pathway is associated with the stimulation of several metabolic pathways, such as glutathione synthesis [180,182], fatty acid and lipid biosynthesis [180,182,183,184,185], the pentose phosphate pathway, and the tricarboxylic acid cycle [186] (Figure 2). In both normal and cancer cells, reduced-glutathione (GSH) maintains redox homeostasis to facilitate cell proliferation [180,187,188,189]. The overactivation of Nrf2 expresses genes, such as malic enzyme 1, isocitrate dehydrogenase-1, 6-phosphogluconate dehydrogenase, and glucose-6-phosphate dehydrogenase, which may be involved in the generation of NADPH [190]. NADPH is an essential co-factor that is required for glutathione synthesis [180,182]. Furthermore, the metabolic reprogramming of cell proliferation is a result of the Nrf2 overactivation-mediated expression of several metabolic enzymes (i.e., transketolase, phosphogluconate dehydrogenase, glucose-6-phosphate dehydrogenase, malic enzyme 1, isocitrate dehydrogenase) in cancer cells. These enzymes facilitate the metabolism of glucose in the pentose phosphate pathway, as well as glutamine and the synthesis of purine and amino acids [180]. In addition, Nrf2 upregulates the expression of genes (i.e., prostaglandin reductase-1 in rat hepatocarcinogenesis) that facilitate the fatty acids and lipids metabolism [180,182,183,184,185]. Furthermore, the reduction of the proliferation capacity of Nrf2-dependent and siRNA-mediated adenocarcinomic human alveolar basal epithelial A549 cancer cells with reduced Nrf2 levels suggests that the activation of Nrf2 is necessity to promote A549 cell proliferation [180]. Furthermore, Nrf2 appears to induce the direct carbon influx to the pentose phosphate pathway and the tricarboxylic acid cycle by regulating microRNA miR-1 and miR-206 [186]. Furthermore, direct carbon influx is associated with accelerated metabolic reactions in the pentose phosphate pathway and the tricarboxylic acid cycle, which facilitates cancer cell growth and proliferation [33,186].

Nrf2 is found to be a regulator of the cell cycle and PI3K/AKT signaling, and in ensuring healthy mitochondrial functions and lifespans in facilitating cancer cell proliferation [191]. Reddy and colleagues [192] showed that Nrf2 deficiency arrests the cell cycle progression at the G2/M phase of primary cortical neuron cultures, avoiding further proliferation. Furthermore, the authors suggest that these anti-proliferative effects are related to diminished glutathione levels [192]. Reduced antioxidant activity leads to an inactivation of the PI3K/AKT pathway, which is restored upon glutathione supplementation, confirming the role of antioxidant defense mechanisms in the anti-proliferative effect of the hepatocytes of Nrf2-deficient C57B/SV129 mice [192,193]. Furthermore, the knockdown of Nrf2 in A549 lung carcinoma cells facilitates the cell cycle arrest at the G1 phase, with the reduction of the phosphorylated retinoblastoma tumor suppressor protein [194]. Nrf2 has a role in cancer proliferation in pancreatic cancer as well [195]. Nrf2 modulates mRNA translation in murine pancreatic organoids by preventing the oxidation of mRNA translational regulatory proteins (i.e., pyruvate kinase PKM, elongation factor 2, 40S ribosomal protein S2, 40S ribosomal protein S4, and valine-tRNA ligase) [195]. Further, the deficient state of Nrf2 (shNrf2) in SUIT-2 pancreatic cancer cells and murine pancreatic organoids has also been shown to impair epidermal growth factor signaling and translational regulatory proteins, which causes impaired mRNA translation, leading to defects in cell proliferation [195]. In addition, activation of the Nrf2/ARE pathway facilitates the proliferation of both malignant and non-malignant cells by its effects on the function and lifespan of mitochondria, which promote healthy aging [191]. The smooth functioning of mitochondria is assured through increasing substrate availability for mitochondria through the activation of Nrf2, which increases mitochondrial membrane potential, ATP levels, rate of respiration, and the efficacy of oxidative phosphorylation. Furthermore, mitochondrial life spans are increased by inducing biogenesis, maintenance, and the removal of the damaged mitochondria while maintaining homeostasis [196,197].

In summary, the activation of the Nrf2/ARE pathway leads to the proliferation of cancer cells by regulating cell cycle proteins, PI3K/AKT signaling, the regulation of cellular metabolism, and maintaining mitochondrial health [191]. Therefore, the downregulation of the Nrf2/ARE pathway by inhibiting Nrf2 activation in cancer cells would be ideal for downregulating cancer cell proliferation and progression [194,198].

### 3.1. Flavonoids: Nrf2/ARE Activation in Cancer Cells

Flavonoids promote cancer in preclinical conditions while exerting positive effects on cancer cell survival, growth, and proliferation in vitro, by activating the Nrf2/ARE pathway through several different mechanisms (Table 2) (Figure 2). Luteolin activates the Nrf2/ARE pathway in multiple human and murine cancer cell lines at several different stages. Studies show not only that luteolin increases the activation of the Nrf2/ARE pathway; it also induces the upregulation of downstream Nrf2/ARE-targeted molecules. Luteolin increases the mRNA and protein levels of Nrf2 in human hepatocellular carcinoma HepG2 cells [65,199]. The inductive effects of luteolin on HepG2 continue further downstream, as evidenced by an increased expression of HO-1 at transcriptional and translational levels [65]. The treatment of another human colorectal cancer cell line (Caco-2) with luteolin increases the nuclear translocation of Nrf2 and increases the GSH level at a downstream level [72]. However, the tested concentrations of luteolin in HepG2 and Caco-2 cells are higher than the bioavailable luteolin from a human diet [199]. Pandurangan and co-workers in 2014 reported a similar effect of luteolin on Nrf2 in a rodent model (azoxymethane-induced colorectal cancer) of BALB/c male mice, with the further activation of GST as well [200]. Furthermore, the tested concentration that activates Nrf2/ARE in BALB/c mice is much lower than concentrations that are toxic to mice upon intraperitoneal administration [201]. Apigenin increases the mRNA and protein expression of Nrf2 in HepG2 human hepatocellular carcinoma cells with oxidative stress [65]. The inductive effects of apigenin on HepG2 continue further downstream, as evidenced by an increased expression of HO-1 at the transcriptional and translational levels [65]. Furthermore, the apigenin-mediated upregulation of Nrf2/ARE in HepG2 is associated with the activation of PI3K /Akt and ERK1/2 signaling [65]. However, the tested concentrations of apigenin in HepG2 cells are higher than the reported physiological concentrations of apigenin in humans [65,101,202].

Myricetin activates Nrf2 in HepG2 human hepatocellular carcinoma cells through canonical activation, via modifying the Keap1 protein [203]. These modifications lead to unchanged levels of Keap1 but inhibit Nrf2 ubiquitination through interfering with the CUL3–Keap1–Nrf2 complex, which activates Nrf2 [203]. Furthermore, myricetin upregulates Nrf2 nuclear translocation following Nrf2 activation in HepG2 [203]. The inductive effects of myricetin on HepG2 continue further downstream, as evidenced by an increased expression of HO-1 at the translational level [203]. However, the bioavailability of tested concentrations of myricetin through human dietary intake is mostly unknown.

Quercetin activates the Nrf2/ARE pathway in chronic high glucose-induced human neuroblastoma cells (SH-SY5Y), with oxidative stress, through sustained phosphorylation in Nrf2 by PKC [123]. The inductive effect of quercetin continues further downstream on SH-SY5Y cells via the upregulation of the nuclear localization of Nrf2 and the expression of phase 2 detoxifying enzyme glyoxalase 1 at transcriptional and translational levels, in addition to increasing its enzymatic activity [123]. Furthermore, quercetin shows similar upregulations of Nrf2 nuclear translocation in *t*-BHP-induced HepG2 cells with oxidative stress [204]. However, the tested concentrations of quercetin in SH-SY5Y and HepG2 cells are higher than bioavailable quercetin from a human diet [110].

Hesperetin, the aglycone of hesperidin, activates the Nrf2/ARE pathway in LPS-induced murine macrophage Raw 264.7 cells [205]. Hesperetin increases the degradation of the Keap1 protein and Nrf2 nuclear translocation in Raw 264.7 [205]. The inductive effects of hesperetin continue further downstream on Raw 264.7 cells, as evidenced by an increased expression of HO-1 at translational levels [205]. Furthermore, the tested concentrations of hesperetin in Raw 264.7 cells are much higher than the physiologically bioavailable hesperetin in human subjects derived from the diet [206]. Neohesperidin dihydrochalcone reduces Nrf2 ubiquitination through the canonical activation of Nrf2 by modifying the Keap1 protein in CCl_4_-induced HepG2 cells with oxidative stress [207]. Furthermore, modifications observed in Keap1 may have interfered with the CUL3–Keap1–Nrf2 complex, which reduces Nrf2 ubiquitination [203,207]. Further, the inductive effects of neohesperidin dihydrochalcone on HepG2 continue further downstream upon Nrf2 activation, as evidenced by Nrf2 nuclear localization and the increased expression of HO-1 and NQO-1 at the translational level [207]. The expression of downstream target proteins (HO-1 and NQO-1) in HepG2 cells depends on the increased levels of phosphorylated p38 and JNK signaling molecules [207]. However, the bioavailability of neohesperidin dihydrochalcone from a human diet is mostly unknown, due to a lack of studies on the bioavailability of neohesperidin in clinical studies.

Genistein and daidzein, two major isoflavones of soybean, upregulate quinone reductase at the transcriptional level and its activity in Hepa-1c1c7 murine hepatoma cells at human physiological concentrations [208]. Furthermore, several other flavonoids belong to flavan-3-ol, and chalcones show their potential in activating the Nrf2/ARE pathway in different cancer models (Table 2).

Overall, most of the flavonoids tested have exhibited their potential in activating the Nrf2/ARE pathway in different cancer models, either under induced or non-induced conditions. However, the exact molecular mechanism of the activation of the Nrf2/ARE pathway by these flavonoids in cancer cells is yet to be explored.

### 3.2. Flavonoids: Nrf2/ARE Inhibition in Cancer Cells

Identification of specific flavonoids that inhibit the Nrf2/ARE pathway in cancer cells would be interesting for exploring their applications in cancer treatment. Interestingly, three flavones, luteolin, apigenin and chrysin, were found to be effective in inhibiting the Nrf2/ARE pathway in different cancer cell lines [60,61,62,63,68,209].

Luteolin inhibits the Nrf2-ARE pathway and downregulates ARE-driven enzymes such as gamma-glutamylcysteine ligase and HO-1 in opisthorchiasis-associated cholangiocarcinoma KKU-100 cells [60]. Luteolin induced these effects, leading to an elevation of superoxide radical levels followed by the mitochondrial depolarization-mediated apoptosis of KKU-100 cells [60]. Another study shows that luteolin reduces the mRNA levels of phase 2 drug detoxifying enzymes, such as HO-1, NQO-1, aldo-keto reductases1 C1 and C2 (AKR1C), glutamate-cysteine ligase catalytic subunit (GCLC), and multidrug resistance-associated protein (MRP) 2 in human cancer cells, A549, siGFP-C5 (a stable lung cancer cell line developed from A549 cells by transfecting pRS-GFP), Caco-2 cells, and human breast cancer MCF7 cells [61]. Interestingly, luteolin sensitizes siGFP-C5 to oxaliplatin, bleomycin and doxorubicin by reducing the IC_50_ values of these chemotherapeutic drugs that are used to treat lung cancer. However, the tested concentrations of luteolin in KKU-100, A549, siGFP-C5, Caco-2 and MCF7 are higher than the physiologically available luteolin in humans through the diet [199].

Apigenin inhibits the Nrf2/ARE pathway in doxorubicin-resistant hepatocellular carcinoma BEL-7402/ADM and HepG2 cancer cells [62,68]. Apigenin inhibits Nrf2 at the mRNA level, and phase 2 detoxifying enzymes at the protein and mRNA levels in BEL-7402/ADM cells [62]. Furthermore, apigenin sensitizes doxorubicin-resistant BEL-7402/ADM cells to doxorubicin by reducing the IC_50_ value of doxorubicin [62,63]. Furthermore, these observations are associated with the improved cellular uptake of doxorubicin with the downregulation of MRP5 gene expression and the downregulation of the PI3K/Akt pathway by reducing phosphorylated Akt, leading to reduced Nrf2 nuclear translocation [62]. Furthermore, the intraperitoneal administration of apigenin significantly reduces the growth of BEL-7402 tumors transplanted into male BALB/c mice without inducing hepatotoxicity [62]. Interestingly, the co-administration of apigenin and doxorubicin in BALB/c male mice shows a greater reduction of BEL-7402 tumor size than the apigenin or doxorubicin treatment alone [62]. Apigenin facilitates the inhibition of cell growth and the ROS-mediated apoptosis of HepG2 cells [68]. Further, apigenin reduces the mRNA levels of antioxidant defense genes (GSH and GPx) in HepG2, as well as the activity of GSH and GPx [68].

The inhibitory effect of chrysin on the Nrf2/ARE pathway in several cancer cells has been shown. Chrysin reduces the mRNA expression of Nrf2, MRP1, NQO-1, and HO-1 in breast cancer MCF7 cells [209]. Chrysin sensitizes MCF7 cells to doxorubicin, and the co-treatment with chrysin and doxorubicin increases the apoptotic MCF7 cell population when compared to chrysin or doxorubicin treatment alone [209]. Similar to apigenin, chrysin downregulates the expression of Nrf2 and phase 2 detoxifying enzymes in doxorubicin-resistant hepatocellular carcinoma BEL-7402/ADM cells [63]. Further, the chrysin-mediated downregulation of the Nrf2/ARE pathway is associated with the inhibition of PI3K/Akt and ERK signaling, and the increased uptake of doxorubicin upon inhibition of MRP5 by BEL-7402/ADM cells [63]. Furthermore, chrysin sensitizes BEL-7402/ADM cells to doxorubicin by reducing the IC_50_ of doxorubicin [63]. However, the reported effective concentrations of chrysin in MCF7 and BEL-7402/ADM cell lines may not be achievable through a human diet or supplementation, due to the low bioavailability of oral administration [210].

**Table 2:** Flavonoids: Nrf2/ARE regulation in cancer cells.

Sub-Group	Compound	Effective Concentration/Concentration Range	Model	Mode of Action	Reference
Flavone	Luteolin	30 µM	Human colorectal carcinoma HCT116 cells	↑ mRNA expression of Nrf2. ↑ Nrf2 protein.	[211]
		10–15 µM	Human epithelial colorectal adenocarcinoma Caco-2 cells	↑ Nrf2 nuclear translocation. ↑ GSH levels.	[72]
		1.2 mg/kg body weight/day intraperitoneal injection once a week for 3 weeks	Colorectal cancer in Balb/C mice	↑ Nrf2 and GST proteins.	[200]
		1.5–6.25 µM	Human hepatocellular carcinoma HepG2 cells	↑ mRNA and protein expression of Nrf2 and HO-1 via PI3K/Akt.	[65]
		1–10 µM	Human epithelial colorectal adenocarcinoma Caco-2 cells	↓ mRNA expression of Nrf2, HO-1, NQO-1, aldo-keto reductases1C1 and C2 (AKR1C), glutamate-cysteine ligase catalytic subunit (GCLC), and multidrug resistance-associated protein (MRP) 2.	[61]
		5–10 µM	Human alveolar basal epithelial adenocarcinoma A549 cells	↓ mRNA expression of HO-1, NQO-1, AKR1C, GCLC, and MRP2. ↓ Nrf2 protein.	[61]
		1–10 µM	Human breast cancer MCF7 cells	↓ mRNA expression of Nrf2, HO-1, NQO-1, AKR1C, GCLC, and MRP2.	[61]
		25 µM	Opisthorchiasis-associated cholangiocarcinoma KKU-100 cells	↓ Nrf2, gamma-glutamylcysteine ligase and HO-1 proteins	[60]
	Chrysin	10–20 µM	Hepatocellular carcinoma BEL-7402/ADM cells	↓ mRNA and protein expression of Nrf2, HO-1, MRP5, and aldo-keto reductase family 1 member B10 (AKR1B10)	[63]
		20 µM	Human breast cancer MCF7 cells	↓ mRNA expression of Nrf2, MRP1, NQO-1, and HO-1.	[209]
	Apigenin	1.56–6.25 µM	Human hepatocellular carcinoma HepG2 cells	↑ mRNA expression of Nrf2. ↑ Nrf2 and HO-1 proteins. ↑ mRNA expression of Nrf2 and HO-1. Activates PI3K/Akt and ERK1/2 signaling.	[65]
		10–20 µM	Hepatocellular carcinoma BEL-7402/ADM cells	↓ mRNA expression of Nrf2. ↓ mRNA and protein expression of HO-1, MRP5, and AKR1B10.	[62]
		100 µM	Human hepatocellular carcinoma HepG2 cells	↓ mRNA expression of GSH and GPx. ↓ GSH and GPx activity.	[68]
	Tangeretin	20 µM	Human hepatocellular carcinoma HepG2 cells	↑ Nrf2 nuclear translocation ↑mRNA and protein expression of HO-1 and NQO-1.	[212]
Flavonol	Myricetin	10–40 µM	Human hepatocellular carcinoma HepG2 cells	Activates Nrf2 by modifying Keap1 protein. ↓ Nrf2 ubiquitination. ↑ Nrf2 nuclear translocation. ↑ ARE binding ability. ↑ Nrf2 protein levels but not Keap1. ↑ protein expression of HO-1.	[203]
	Quercetin	10 µM	Human hepatocellular carcinoma HepG2 cells	↑ Nrf2 nuclear translocation. ↑ARE binding activity of Nrf2.	[204]
		10 µM	Human neuroblastoma SH-SY5Y cells	↑ Nrf2 nuclear translocation. ↑ Nrf2 phosphorylation via PKC activation. ↑ protein and mRNA expression of glyoxalase-1. ↑ glyoxalase-1 activity	[123]
	Rutin	44 µM	Human epithelial colorectal adenocarcinoma Caco-2 cells	↑ mRNA expression of Nrf2, HO-1 and NQO-1 without changing Keap1 mRNA levels.	[140]
Flavanone	Hesperetin	40 µM	Murine macrophage Raw 264.7 cells	↑ Nrf2 nuclear translocation. ↑ degradation of Keap1. ↑ protein expression of HO-1	[205]
	Neohesperidin dihydrochalcone	30 µM	Human hepatocellular carcinoma HepG2 cells	↑ Keap1 modifications ↑ Nrf2 nuclear translocation. ↑ Nrf2 ARE binding ability. ↓ Nrf2 ubiquitination. ↑ phosphorylated JNK and p38 dependent protein expression of HO-1 and NQO-1.	[207]
	Naringenin	20–80 µM	Human neuroblastoma SH-SY5Y cells	↑ Nrf2 nuclear translocation. ↑ GSH protein ↑ protein expression of HO-1	[112]
Flavan-3-ol	Epicatechin	10 µM	Human hepatocellular carcinoma HepG2 cells	↑ Nrf2 phosphorylation. ↑ Nrf2 nuclear translocation.	[213]
	Morin	5–10 µM	Rat insulinoma INS-1E cells	↑ Nrf2 phosphorylation. ↑ Nrf2 nuclear translocation.	[214]
Isoflavones	Daidzein	5 µM	Murine hepatoma Hepa-1c1c7 cells	↑ mRNA expression of quinone reductase. ↑ quinone reductase activity. ↑ ARE binding ability	[208]
	Genistein	5 µM	Murine hepatoma Hepa-1c1c7 cells	↑ mRNA expression of quinone reductase. ↑ quinone reductase activity. ↑ ARE binding ability.	[208]
Chalcone	Phloretamide	20 µM	Human hepatocellular carcinoma HepG2 cells	↑ Nrf2 nuclear translocation. ↑mRNA expression of GST and NQO-1.	[215]

Abbreviations—HCT116: human colorectal carcinoma cell line; Caco-2: human epithelial colorectal adenocarcinoma cell line; KKU-100: opisthorchiasis-associated cholangiocarcinoma cell line; A549: human alveolar basal epithelial adenocarcinoma cell line; MCF7: human breast cancer cell line; BEL-7402/ADM: hepatocellular carcinoma cell line; HepG2: human hepatocellular carcinoma cell line; SH-SY5Y: human neuroblastoma cells; Raw 264.7: murine macrophage cell line; INS-1E: rat insulinoma cell line; Hepa-1c1c7: murine hepatoma cell line; GST: glutathione S-transferase; NQO-1: NAD(P)H quinone dehydrogenase 1; HO-1: heme oxygenase 1; AKR1C: aldo-keto reductases1 C1 and C2; GCLC: glutamate-cysteine ligase catalytic subunit; MRP: multidrug resistance-associated protein; AKR1B10: aldo-keto reductase family 1 member B10; PKC: protein kinase C; ERK: extracellular signal-regulated protein kinase; PI3K/Akt: phosphorylation of phosphatidylinositol 3-kinase/protein kinase B; JNK: N terminal kinase; Keap1: Kelch-like ECH-associated protein 1; Nrf2: Nuclear factor erythroid 2 p45 (NF-E2)-related factor; ↑ increase; ↓ decrease.

In cancer cells, flavonoids can activate the Nrf2/ARE pathway, subsequently leading to the restoration of redox homeostasis via the detoxification of xenobiotics, drug transportation, and the neutralization of reactive species. This thereby facilitates cancer cell survival and cancer promotion. The expression of several ARE-driven genes facilitates cancer cell proliferation by facilitating metabolic reactions such as glutamine synthesis [180,182], NADPH and glucose metabolism in the pentose phosphate pathway [180,182,183,184,185] and the tricarboxylic acid cycle, in addition to fatty acid and lipid metabolism [180,182,183,184,185], and purine and amino acids synthesis [180,182,183,184,185]. Further, the activation of the Nrf2/ARE pathway was achieved, with influential crosstalk facilitated by several flavonoids in between other signaling molecules and the Nrf2/ARE pathway. Therefore, it will be beneficial if the Nrf2/ARE pathway can be inhibited by certain flavonoids in cancer cells [40].

1: Luteolin; 2: Apigenin; 3: Tangeretin; 4: Myricetin; 5: Quercetin; 6: Rutin; 7: Hesperetin; 8: Neohesperidin dihydrochalcone; 9: Epicatechin; 10: Naringenin; 11: Phloretomide; 12: Chrysin; 13: Daidzein; 14: Genistein; 15: Naringenin; 16: Morin.

Keap1: Kelch-like ECH-associated protein 1; Nrf2: Nuclear factor erythroid 2 p45 (NF-E2)-related factor; sMaf: Small musculoaponeurotic fibrosarcoma protein; ARE: Antioxidant response element; GSH: Glutathione; SOD: superoxide dismutase; CAT: Catalase; GPx: Glutathione peroxidase, FA: fatty acid; AA: Amino acid; NADPH: nicotine adenine dinucleotide phosphate; TCA: tricarboxylic acid.

(The figure was adapted from Wu et al., 2019 [33])

## 4. Flavonoids and Nrf2/ARE: A Friend or Foe

The role of flavonoids in the activation of the Nrf2/ARE pathway has led to controversial debates over their dual roles in cancer prevention (in normal cells) and cancer promotion (in cancer cells) [33]. Certain flavonoids have the potential to promote or inhibit cancer/cancer cell survival, growth, and proliferation by regulating the Nrf2/ARE pathway differently [65,93].

As previously described, luteolin exhibits dual roles in cancer cell growth in vitro and cancer promotion in vivo, in addition to cancer prevention in vitro and in vivo. Luteolin activates Nrf2/ARE in cancer models, such as human colorectal carcinoma HCT116, Caco-2, and HepG2 [65,72,200]. In comparison, luteolin activates the Nrf2/ARE pathway in H9C2 and mouse testis Sertoli TM4 cells to protect cells from oxidative stress-mediated apoptosis [93,121]. Luteolin reduces LPS-induced severe acute pancreatitis in ICR mice [193]. Furthermore, luteolin upregulates the Nrf2/ARE pathway in ICR mice under normal physiological conditions, and Sprague-Dawley rats with intracerebral hemorrhage [51,131]. Further, luteolin mediates the upregulation of Nrf2 at both the mRNA and protein levels in both non-cancer (H9C2) and cancer models (HCT116, HepG2 cells, and azoxymethane-induced colorectal cancer induced BALB/c mice) [65,72,93,200]. Furthermore, luteolin facilitates Nrf2 nuclear translocation in non-cancer (TM4 cells, liver cells of ICR mice, and basal ganglia cells of Sprague-Dawley rats) and cancer (HepG2 and Caco-2 cells) models [51,65,72,121,131]. Furthermore, luteolin is effective in expressing the HO-1 protein in ICR mice, Sprague-Dawley rats with intracerebral hemorrhage, and human HepG2 cancer cells [51,65,131].

Interestingly, the upregulation of Nrf2 at both the mRNA and protein levels in HCT116 cells is associated with cancer prevention through the reducing/reversing of epigenetic modifications (CpG methylation) in the Nrf2 gene promoter, and blocking HCT116 cell proliferation and transformation [211]. Furthermore, further exposure to azoxymethane in azoxymethane-induced colorectal cancer BALB/c mice shows that the upregulation of GST in the liver and colon by luteolin detoxifies azoxymethane to avoid the further initiation of normal cells to colorectal cancer cells [200]. However, cancer-initiated cells may survive with the upregulation of the Nrf2/ARE pathway and lead to cancer progression, even though exposure to carcinogens is dealt with, as the activation of Nrf2/ARE can lead to cancer cell survival and proliferation [33,200]. Further, low concentrations of luteolin significantly increase cell viability in HepG2 cancer cells, which may have the potential to facilitate cancer progression [65]. In contrast, luteolin exhibits inhibitory effects on KKU-100, siGFP-C5, MCF7, Caco-2, and A549 cells by downregulating the Nrf2/ARE pathway [61]. However, luteolin exhibits hormetic effects in MCF7, Caco-2, siGFP-C5, and A549 cells in a dose-dependent manner. At low concentrations, luteolin increases the expression of HO-1, NQO-1, AKR1C, GCLC, and MRP2 genes (protein and mRNA levels) in MCF7, Caco-2, siGFP-C5, and A549 cells, and high concentrations of luteolin significantly reduce the gene expression. Furthermore, the luteolin-induced downregulation of the expression of ARE-driven enzymes increases the sensitivity of siGFP-C5 cells, which is transfected with pRS-GFP (expresses siRNA against GFP mRNA; siNrf2-C27), to chemopreventive drugs such as oxaliplatin, bleomycin, and doxorubicin [61]. Furthermore, a study carried out by Yang and colleagues (2014) showed that low doses of luteolin encourage the upregulation of cellular GSH, and comparatively higher concentrations of luteolin encourage the reduction of GSH content in Caco-2 cell line [72]. Thus, luteolin may have the potential to exert anti-cancer effects via its hormetic effects.

Apigenin (flavone) also showed dual roles in vitro with respect to cancer prevention and cancer cell survival, growth, and proliferation in different non-cancer (ARPE-19) and cancer (HepG2) cell lines, respectively [65,95,100]. The expression of Nrf2 at both the mRNA and protein levels is commonly seen in ARPE-19and HepG2 cells [65,95]. In ARPE-19 cells, apigenin alleviates *t*-BHP-induced oxidative stress with the increased activities of antioxidant defense enzymes (SOD, CAT, and GPx), and reduces cell apoptosis by restoring normal physiological concentrations [95]. Further, at the tested low-concentrations, apigenin alone significantly improved cell viability in HepG2 cancer cells [65]. Thus, along with the Nrf2/ARE activation, cell growth and cell proliferation have been increased in HepG2 cells at the tested concentrations (1.5-6.25 µM) [65]. Contrarily, the hormetic effect of apigenin is evident, as higher concentrations (100 µM) inhibit cancer cell survival in HepG2 cancer cells via the excessive generation of ROS-mediated apoptosis [68]. Furthermore, the possible use of low doses of apigenin in treating hepatocellular carcinoma has been validated in both BEL-7402/ADM cells (10–20 µM) and BEL-7402 transplanted BALB/c mice (50 mg/kg), with the inhibition of cancer cells and tumors in the animal model, respectively. Furthermore, apigenin sensitizes hepatocellular carcinoma cells (BEL-7402/ADM) to chemotherapeutic drugs such as doxorubicin, indicating the promising synergistic effects of the co-treatment of apigenin and chemotherapeutic drugs [62].

Chrysin activates the Nrf2/ARE pathway in rat hepatocytes to attenuate *t*-BHP-induced oxidative stress, and restores redox homeostasis [124]. Furthermore, the inhibitory effects of apigenin were observed in both cancer (BEL-7402/ADM and MCF7) and non-cancer (HUVEC) cells [124]. At the same time, chrysin sensitizes MCF7 and BEL-7402/ADM cancer cells to doxorubicin, indicating the potential use of chrysin along with chemotherapy to treat breast cancer and hepatocellular carcinoma after further validation in pre-clinical and clinical studies [63,209]. However, chrysin acts as an inhibitor of Nrf2 in HUVECs, as it downregulates the protective effects of hydrogen. Molecular hydrogen protects HUVECs from anti-senescence by activating the Nrf2/ARE pathway [216]. Hydrogen treatment upregulates the phosphorylated Nrf2 and phase 2 detoxifying enzymes, such as HO-1 and NQO-1, at protein levels [216]. However, chrysin treatments inhibit the antioxidant and anti-senescence activities of hydrogen by downregulating HO-1 and NQO-1 proteins and increasing DNA damage, as shown by the significantly increased 8-hydroxydeoxyguanosine level in HUVEC cells [216]. Thus, chrysin exhibits its potential to inhibit the Nrf2/ARE pathway in HUVECs under normal physiological conditions, while activating it in non-cancer (rat hepatocytes) cells with induced conditions.

Baicalin and its aglycone baicalein activate the Nrf2/ARE pathway in non-cancer experimental models [94,117,120]. Baicalin activates the Nrf2/ARE pathway in both human liver L-02 and murine H9C2 cells, and in Sprague-Dawley rats. Further, baicalin upregulates Nrf2 at the translational level in H9C2 and the brain tissues of Sprague-Dawley cells [94,97]. Furthermore, baicalin protects H9C2 cells from hypoxia-aroused apoptosis, and reduces early brain injury in Sprague-Dawley rats after subarachnoid hemorrhage [97]. Further, the inductive effects of both baicalein and baicalin show that human liver L-02 cells can be protected from acetaminophen-induced oxidative stress and liver cell injury [117]. In another study, Dong and colleagues (2020) showed that baicalein upregulates Nrf2 nuclear translocation in high glucose-induced human liver L-02 (synonym: HL-7702) cells, and the liver cells of T2DM Kunming mice, and reduces oxidative stress in both the L-02 and the liver cells of T2DM Kunming mice, in addition to the reduction of L-02 cell apoptosis [120].

Quercetin, a common flavonol aglycone, exhibits the activation of the Nrf2/ARE pathway in both cancer (HepG2 and SH-SY5Y) and non-cancer cells (HaCaT and BJ foreskin fibroblasts, IEC-6 and HUVECs), and in animal models of broiler chicken [102,108,111,123,155,204]. Quercetin exerts inductive effects on Nrf2 nuclear translocation in HepG2, SH-SY5Y, broiler chicken, and IEC-6 cells. Further, the upregulation of the Nrf2 protein is common among non-cancer cells (HUVECs, HaCaT, and BJ foreskin fibroblasts) and in broiler chickens. The quercetin-mediated induction of metallothionein and the activation of the Nrf2/ARE pathway in HepG2 protects from *t*-BHP-induced oxidative stress [204]. Furthermore, the expression of Nrf2 downstream protein metallothionein is associated with protecting liver cells from acute heavy metal toxicity through binding heavy metals intracellularly [204,217,218]. Therefore, the hepatoprotection exerted by the quercetin-mediated induction of metallothionein in HepG2 cells may be partly due to the binding of metallothionein with metals present in the liver (i.e., Fe) that have the potential to participate in Fenton reaction and Haber–Weiss reactions, to generate further ROS [204,219]. Furthermore, the quercetin-mediated activation of the Nrf2/ARE pathway protects SH-SY5Y cancer cells from chronic high glucose-induced oxidative cell injury, and exerts neuroprotection in addition to improving cell viability [123]. Therefore, the protective effects of quercetin with the induction of the Nrf2/ARE pathway may facilitate cancer cell survival and proliferation in HepG2 and SH-SY5Y cells. In contrast, quercetin activates the Nrf2/ARE pathway in HaCaT and BJ foreskin fibroblasts under normal physiological conditions, without any oxidative inducers [102]. Furthermore, quercetin activates Nrf2/ARE with the presence of oxidative stress inducers in hydrogen peroxide-induced IEC-6 and HUVECs cells, and in LPS-induced broiler chickens, and restores normal physiological conditions [108,111,155].

Myricetin and rutin are another two flavanols that activate the Nrf2/ARE pathway in both cancer and non-cancer experimental models [132,140,203]. Myricetin treated HepG2, and normal Kunming mice show similar mechanisms of Nrf2/ARE activation in terms of the upregulation of Nrf2 nuclear translocation. Furthermore, myricetin shows that it can activate the Nrf2/ARE pathway in HepG2 cells and Kunming mice without any oxidative inducers [132,203]. Furthermore, rutin activates the Nrf2/ARE pathway, leading to a subsequent upregulation of HO-1 and NQO-1 at the transcriptional level in human keratinocyte HaCaT cells and colorectal cancer cells (Caco-2), in an oxidative inducer-independent manner [140]. Further, rutin did not increase ROS production in HaCaT and Caco-2 cells, which suggests that rutin alone acts as an activator of the Nrf2/ARE pathway without increasing oxidative stress [140].

Naringenin activates the Nrf2/ARE pathway in 6-OHDA-induced SH-SY5Y cancer cells and male C57BL/6 mice, attenuating oxidative stress [112]. Furthermore, naringenin protects SH-SY5Y cancer cells against 6-OHDA-induced oxidative stress-mediated apoptosis [112]. Further, the inductive effects of naringenin continue as it activates the Nrf2/ARE pathway in normal Sprague-Dawley neuron cells in vitro in the absence of oxidative inducers, and reduces oxidative stress-mediated mitochondrial dysfunctions [103]. Furthermore, the upregulation of HO-1 at the translational level was observed in Sprague-Dawley rat neuron cells and 6-OHDA-induced SH-SY5Y cells [103,112]. Furthermore, low concentrations of naringenin elevate ROS production in Sprague-Dawley neurons and, therefore, the activation of the Nrf2/ARE pathway in Sprague-Dawley neurons upon naringenin treatment is partly attributed to ROS production [103].

Epicatechin, a flavan-3-ol, facilitates the Nrf2/ARE pathway by upregulating Nrf2 nuclear translocation in primary mouse astrocytes of wild type (WT) and Nrf2 knock-out (KO) C57BL/6 mice, and in HepG2 cells [213]. Furthermore, epicatechin protects primary mouse astrocytes from hemoglobin-induced oxidative stress, and improves HepG2 cell survival and cell proliferation by activating the Nrf2/ARE pathway [122]. Even though genistein activates the Nrf2/ARE pathway against cerebral ischemia-induced oxidative stress, in Sprague-Dawley rats and normal murine hepatoma Hepa-1c1c cells without oxidative inducers similar mechanisms of activation were not observed [138,208].

Overall, flavones (i.e., luteolin, apigenin, chrysin), flavonols (i.e., myricetin, quercetin, and rutin), flavan-3-ols (i.e., epicatechin), flavanones (i.e., naringenin), and isoflavones (i.e., genistein) exhibit dual roles in the activation of the Nrf2/ARE pathway in both cancer and normal cells/experimental animals. Therefore, flavonoids can be considered as a double-edged sword, or a friend and a foe, as the same compounds have the potential to act as a chemopreventive agent as well as a compound that may have the potential to facilitate cancer cell survival, growth and proliferation. Most of the studies related to flavonoids are reported in the in vitro settings, and there are no clinical data to comment on the dual action of flavonoids in the activation/inhibition of the Nrf2/ARE pathway in cancer and normal cells.

## 5. Conclusions and Future Directions

The effect of flavonoids on the regulation of the Nrf2/ARE pathway in non-cancer (Figure 1) and cancer (Figure 2) is mostly reported using in vitro experimental models. Only a few in vivo studies have been conducted to date. Therefore, the influence of the dietary intake of flavonoids on the Nrf2/ARE pathway associated with physiological benefits is mostly unknown. Based on the reported in vitro and in vivo investigations, it is suggested that if efficacious concentrations of flavonoids can be reached in targeted tissues, flavonoids could contribute to the management of oxidative homeostasis, and thus reduce the risk of cancer. Further, the observed cytotoxicity and anti-proliferative activity in cancer cells of luteolin, apigenin and chrysin should be further validated using in vivo experiments. When considering the bioavailability of flavonoids, most of the effective concentrations of flavonoids that are reported in cell-based assays might not be physiologically relevant [220]. The physiological concentrations that can be achieved through the human diet/oral administration are much lower than most of the tested concentrations in normal (5-400 µM) and cancer (1.5–80 µM) cell lines [221]. For example, the maximum peak plasma concentration of chrysin in healthy human volunteers, after consumption of two 200 mg capsules containing chrysin in the morning following overnight fasting, is 0.01–0.06 nM (3–16 ng/mL) [210]. Therefore, the tested high concentrations of chrysin will never be realized in vivo or in clinical studies in order to have benefits upon oral administration. Further, the peak plasma concentration of chrysin metabolite, chrysin sulfate, is 30 times higher than the administered chrysin concentration in healthy human volunteers (two capsules of 200 mg chrysin via oral administration), despite this being in low concentrations [210]. During phase 2 metabolism, flavonoids undergo sulfation, glucuronidation and methylation in the enterocytes of the small intestine and the liver, resulting in different metabolites. Thus, phase 2 metabolism alters the bioavailability and bioefficacy of the parent compound/flavonoid [222,223]. Hence it is ideal for testing the effectiveness of dietary flavonoids within the physiologically relevant concentration range, in addition to evaluating the effectiveness of metabolites of dietary flavonoids at physiologically relevant concentrations in both in vitro and in vivo experiments. Further, flavonoids such as chrysin have been shown exert positive effects on cancer cell inhibition at comparatively low concentrations, through synergistic effects influenced by one or more other phytochemicals and in low concentrations, compared to acting alone. Therefore, the study of the combinatorial effects of multiple flavonoids/flavonoids and phytochemicals in in vitro, pre-clinical and clinical conditions can be encouraged, which may have the ability to exert chemopreventive effects at lower concentrations [224,225]. Furthermore, due to the low oral bioavailability of luteolin, apigenin and chrysin, the special emphasis should be given to alternative modes of administration, such as intraperitoneal administration, concerning the potential use of these compounds in cancer treatment, but at safer doses [101,199,210].

A cohort study conducted by Bondonno and colleagues, lasting 23 years and using 56,048 cancer-free participants, has shown that the moderate habitual flavonoid intake from a Danish diet (about 500 mg flavonoids/day) is inversely associated with cancer-related mortality [226]. This association is much stronger among participants who consume tobacco and alcohol [226], suggesting that the benefits of dietary flavonoids are noticeable among high-risk groups of cancer. Similar results were observed in a cohort study conducted using 34,708 postmenopausal women who were former or current smokers. The dietary intake of foods rich in flavanones and proanthocyanins reduced the risk of lung cancer incidence, while the intake of foods rich in isoflavones reduced the risk of overall cancer incidence [227]. Considering the digestive tract cancers, a meta-analysis of 23 studies has shown that an increased dietary intake of flavonoids reduces the risk of gastric cancer in European populations [228]. Furthermore, the risk of colorectal cancer among humans is inversely associated with the increased intake of foods rich in flavonols, flavan-3-ols, anthocyanidins and proanthocyanidins [229]. A meta-analysis of 12 epidemiological studies, including five cohort and seven case-control studies, suggests that the risk of colon cancer can be reduced by a high intake of dietary flavonols, whereas the risk of rectal cancer can be reduced by a high intake of flavones [230]. Furthermore, a population-based case-control study by Gates and colleagues shows that the dietary intake of apigenin reduces the risk of ovarian cancer [231]. In contrast, in a health study of 38,408 women aged ≥45 years, Wang and colleagues did not find a major role of five individual flavonols (quercetin, kaempferol and myricetin) and flavones (apigenin and luteolin), or selected flavonoid-rich foods, in cancer prevention [232]. In another study, total flavonoid, quercetin, myricetin and kaempferol intake was not inversely associated with colorectal cancer risk among 71,976 women and 35,425 men involved in the health profession in the US [233]. The above observed lack of correlation could be due to the low intake of flavonoids in the studied populations. Overall, the reported studies suggest that the dietary intake of flavonoids has the potential to reduce the risk of several cancers in most of the studied populations. However, since there are limited or no promising in vivo and clinical data related to the concern that flavonoids may promote already existing cancer growth through the activation of the Nrf2/ARE pathway, further investigations are warranted.

Furthermore, most of the in vitro studies on the activation of the Nrf2/ARE pathway by different flavonoids have been carried out for limited types of cancer cell lines, i.e., HepG2 and Caco-2 cells. The reported studies reveal that the activation or inhibition of the Nrf2/ARE pathway in cancer or normal cells is dependent on flavonoid structure. Therefore, in the future, it is essential to study the structure of the flavonoid vs. its function using different cell lines with dose–response in mind. Furthermore, in silico molecular docking can be used for predicting the influence of the flavonoid structure’s interaction with various proteins associated with the mechanisms of activation of the Nrf2/ARE pathway. These preliminary studies should proceed for a better understanding of the roles of flavonoids in cancer prevention and treatment, before going towards clinical studies. Single flavonoids have been used extensively in the reported molecular mechanistic studies. This is appropriate in fundamental studies as well as in flavonoid-inspired drug discovery. However, in food systems, flavonoids do not present as individual compounds, but exist as a mixture of compounds along with other types of phytochemicals in a food matrix. These complex compound mixtures present in food systems may interact in unpredictable ways with the patients. Therefore, it is also important to assess well-characterized and standardized natural phytochemical mixtures, crude extracts of flavonoids or flavonoid-rich food in light of their effects on the Nrf2/ARE pathway-related management of certain cancers. As reviewed above, some dietary flavonoids could interfere with conventional anticancer therapies by upregulating Nrf2/ARE, which could lead to cytoprotective mechanisms in malignant cells. Recently, Milkovic and colleagues reviewed the controversy around the pharmacological modulation of Nrf2 in relation to cancer therapy, and emphasized the need for a further investigation into inhibitors of Nrf2, which could be a proto-oncogene of cancer patients [234].

In conclusion, the Nrf2/ARE pathway plays a dual role in cancer management via cytoprotection in both normal and cancer cells. Therefore, dietary flavonoids have the potential to exert either cancer-preventive or cancer-promotive influences, depending on the stage of carcinogenesis. Since certain flavonoids can activate the Nrf2/ARE pathway in cancer cells, flavonoid-rich food or supplements given to cancer patients may promote the disease, or interfere with radiotherapy and many forms of chemotherapeutics. Therefore, properly designed clinical studies are required to validate this presumption.

## Abbreviations

**Akt**	Protein kinase B
**AMPK**	AMP-activated protein kinase
**Cul3**	Cullin 3
**ERK**	Extracellular signal-regulated protein kinase
**GPx**	Glutathione peroxidase
**GSH**	Glutathione
**GSK-3β**	Glycogen synthase kinase 3 beta
**HO-1**	Heme oxygenase 1
**HUVECs**	Human umbilical vein epithelial cells
JNK	*n*- terminal kinase
**Keap1**	Kelch-like ECH associated protein 1
**NADPH**	Nicotine adenine dinucleotide phosphate
**NQO1**	NAD(P)H quinone dehydrogenase 1
**Nrf2/ARE**	Nuclear factor erythroid 2-related factor 2/Antioxidant response element
**ROS**	Reactive oxygen species
**SOD**	Superoxide dismutase
**T2DM**	Type 2 diabetes mellitus
**t-BHP**	tert-butyl hydroperoxide
**p62**	Sequestosome 1
**PERK**	Protein kinase-like endoplasmic reticulum-resident kinase
**PI3K**	Phosphatidylinositol 3-kinase
**PKC**	Protein kinase C
**6-OHDA**	6-hydroxydopamine
